# Nuclear alpha-synuclein accelerates cell senescence and neurodegeneration

**DOI:** 10.1186/s12979-024-00429-0

**Published:** 2024-07-12

**Authors:** Tingfu Du, Guoxiang Li, Qinglan Zong, Haiyu Luo, Yue Pan, Kaili Ma

**Affiliations:** 1 Institute of Medical Biology, Chinese Academy of Medical Sciences and Peking Union Medical College, Kunming, 650118 China; 2 Yunnan Key Laboratory of Vaccine Research Development on Severe Infectious Diseases, Kunming, 650118 China

**Keywords:** Parkinson’s disease, Alpha-synuclein, Senescence, SASP, Inflammatory response, Loss of neurons

## Abstract

**Background:**

The progression of Parkinson’s disease (PD) is related to ageing. The accumulation of nuclear alpha-synuclein (α-syn) may accelerate the occurrence of neurodegenerative diseases, but its role in PD remains poorly understood.

**Methods:**

In the present study, α-syn expression was specifically targeted to the nucleus by constructing an adeno-associated virus (AAV) vector in which a nuclear localization sequence (NLS) was added to the α-syn coding sequence. Virus-mediated gene transfer, behavioural tests, RNA-Seq, immunohistochemistry, western blotting, and quantitative real-time PCR were then performed.

**Results:**

*In vivo* experiments using a mouse model showed that nuclear α-syn increased the severity of the PD-like phenotype, including the loss of dopaminergic neurons concomitant with motor impairment and the formation of α-syn inclusions. These nuclear inclusions contained α-syn species of high molecular weights and induced strong transcriptional dysregulation, especially induced high expression of p21 and senescence-associated secretory phenotype (SASP)-related genes. In addition, the transcriptional alterations induced by nuclear α-syn were associated with gliosis, inflammation, oxidative and DNA damage, and lysosomal dysfunction, and they eventually accelerated neuronal loss and neurodegeneration.

**Conclusions:**

Our results suggest that nuclear α-syn plays a crucial role in PD pathogenesis.

**Supplementary Information:**

The online version contains supplementary material available at 10.1186/s12979-024-00429-0.

## Background

PD is a common chronic neurodegenerative disorder that affects approximately 1% of the population over the age of 65. The clinical manifestations of PD include muscular rigidity, static tremor, bradykinesia, and gait difficulty [1, 2]. PD is an age-related disorder of sporadic or familial origin, with familial cases typically developing later in life. The disease is thought to be the result of a combination of genetic and environmental risk factors, as well as aging [3]. PD reduces the quality of life of patients and seriously affects patient health. However, there is currently no effective treatment for PD because the mechanisms underlying its pathogenesis remain unclear.

α-Syn is a protein that is 140 amino acids in length and it is encoded by the SNCA gene. α-Syn is widely expressed in the nervous system and is involved in the maintenance of normal synaptic function [4–6]. The pathological accumulation of α-syn has been shown to correlate with neurodegenerative diseases such as PD [7, 8]. A study demonstrated that α-syn primarily concentrates in presynaptic nerve terminals [9]. In addition, α-syn can be detected in the nucleus, especially under pathological conditions [10–13]. The localization of α-syn to the nucleus has been found in cell lines, animal models, and brain tissues from PD patients [10, 14–19]. Mutations in α-syn (A30P, A53T, and G51D) correlate with the progression of PD and mutant α-syn is predominantly localized to the nucleus [20, 21]. Some studies have shown that α-syn might be involved in the regulation of gene expression [22, 23]. Most of the genes whose expression is affected by α-syn are involved in the regulation of synaptic functions, mitochondrial damage, cell cycle regulation, lysosomes, and phagosome pathways [8]. The transcriptional regulation exerted by α-syn is potentially mediated through its binding to chromatin [24], histones [25, 26], and DNA [16, 27, 28]. Fink et al. found that α-syn can interact with histones in the nuclei of nigral neurons, which suggests that the nuclear accumulation of α-syn may accelerate the occurrence of neurodegenerative diseases [25]. A previous study found that targeting α-syn to the nucleus promoted toxicity in cultured cells and transgenic *Drosophila* [21]. Pinho et al. found that α-syn in the nucleus promoted its interaction with DNA and influenced gene expression, leading to dysregulation of cell cycle-related gene expression and ultimately to neuronal death [16]. There is no consensus on whether the presence of α-syn in the nucleus is protective or cytotoxic. There is increasing evidence that nuclear α-syn is linked to toxicity and neurodegeneration. However, other studies have reported that nuclear α-syn enhance cellular protection [16, 26]. Thus, α-syn plays an important role in the nucleus, but its role in PD remains poorly understood. Previous studies were primarily based on in vitro experiments. Therefore, it is necessary to further explore the role of nuclear α-syn in the pathogenesis of PD in vivo.

In the present study, we used an AAV vector in which a nuclear localization sequence was added to the coding sequence of α-syn to specifically target this protein to the nucleus. Vectors encoding α-syn with or without a NLS were then injected into mice, and the effects on the pathogenesis of PD were observed. The objective of this study was to determine the function of nuclear α-syn, elucidate the mechanism by which nuclear α-syn affects PD, and further deepen our understanding of the relationship between α-syn and the occurrence of PD.

## Results

### Accumulation of nuclear α-syn increased motor impairment

**Fig. 1:**
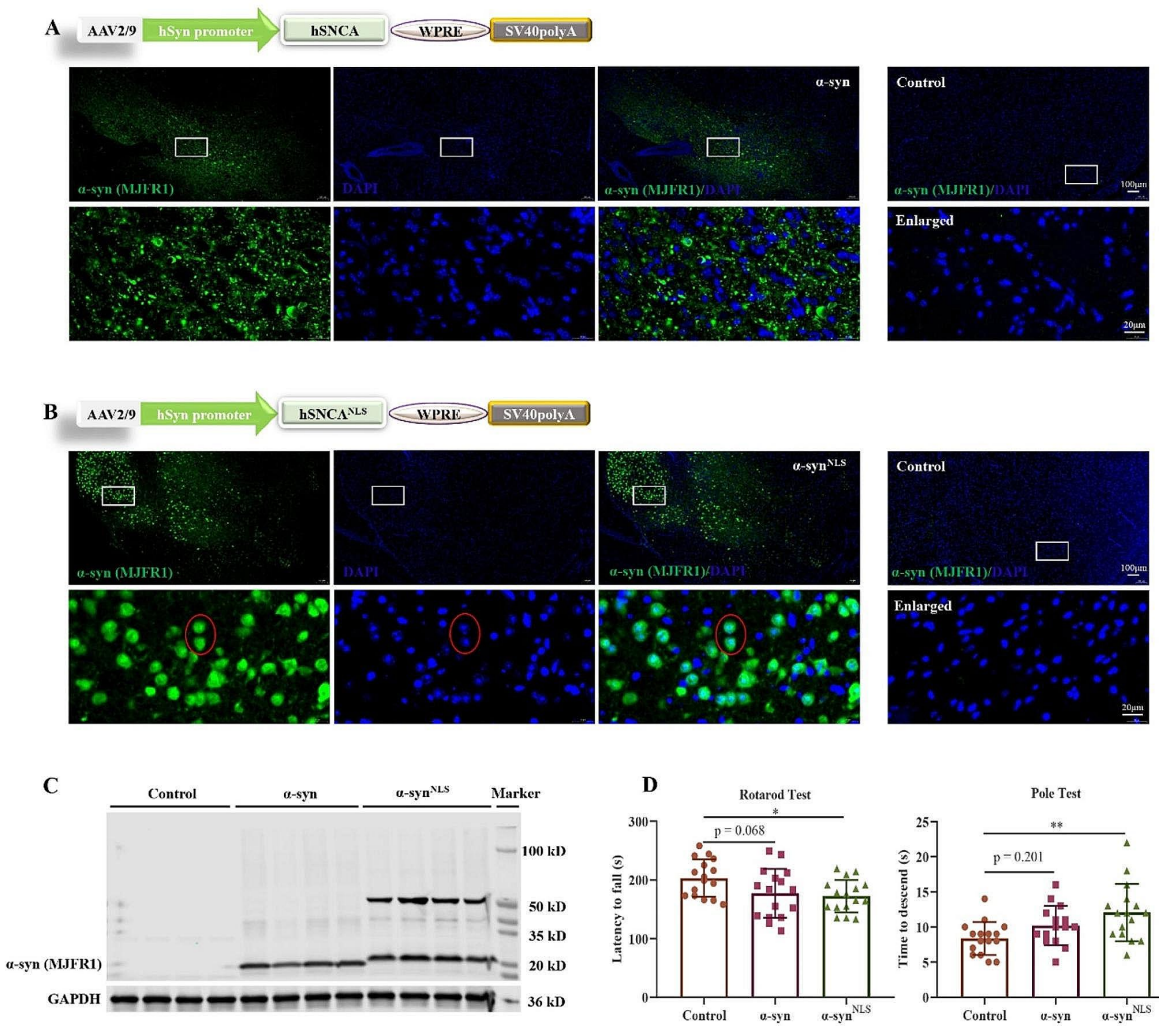
Accumulation of α-syn in the nucleus increased motor impairment in mice (**A, B**) Top panel: Schematic representation of the constructs used in this study; the human α-syn and α-syn^NLS^ genes were subcloned into an AAV2/9 plasmid under the transcriptional regulation of the hSyn promoter (AAV2/9-hSyn-α-syn and AAV2/9-hSyn-α-syn^NLS^), and AAV2/9-hSyn-EGFP served as the control. Bottom panel: IF images showing α-syn expression driven by AAV-α-syn and AAV-α-syn^NLS^ in the SNc one month after injection; *n* = 3 per group. (**C**) The WB results confirmed the overexpression of AAV-α-syn and AAV-α-syn^NLS^. α-syn^NLS^ overexpression resulted in an accumulation of a higher-molecular-weight α-syn species; *n* = 4 per group; the anti-α-syn antibody (clone MJFR1) was used specifically to detect human α-syn. (**D**) Mice overexpressing α-syn (especially in the nucleus) fell off on the faster rotarod test and required more time to descend the pole one month after injection; *n* = 15 per group; *p < 0.05, **p < 0.01. The error bars represent the SD.

The expression of α-syn was assessed using immunofluorescence staining (IF). Widespread α-syn expression was evident on the injection side of the mice. One month after injection, α-syn was primarily expressed in the synapses, whereas α-syn^NLS^ was primarily localized to the neuronal nuclei (Fig. 1A, B). Western blotting (WB) analysis confirmed the overexpression of α-syn in the substantia nigra compacta (SNc) of the treated mice (Fig. 1C) and showed that α-syn^NLS^ accumulated as a species with a molecular weight of approximately 50 kDa. To examine whether α-syn expression altered the behaviour of the treated mice, their motor behaviour was assessed using the rotarod and pole tests. Balance and motor coordination were assessed by comparing the performance of the mice on an accelerating rotarod. Bradykinesia was assessed using the pole test. One month after injection, the mice overexpressing α-syn, especially those overexpressing the nuclear-targeted form of α-syn, fell off the rotarod more quickly and required more time to descent the pole (Fig. 1D).

### PD-like pathology in mice was evident upon the nuclear overexpression of α-syn

**Fig. 2:**
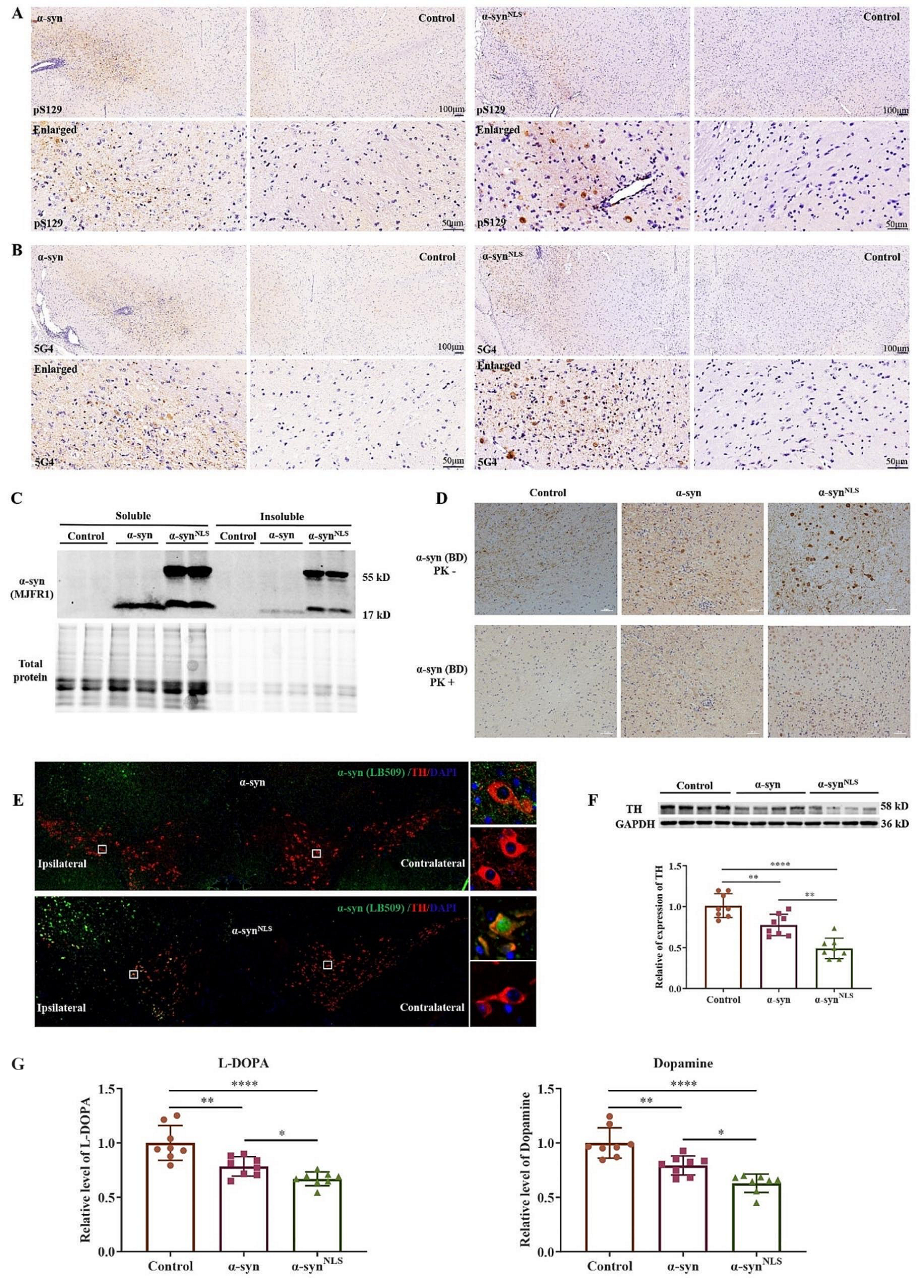
Evident PD-like pathology in mice overexpressing nuclear α-syn (**A**) Phosphorylated α-syn, which was stained with the monoclonal antibody pS129, was highly increased after α-syn overexpression in the SNc; *n* = 3 per group. (**B**) α-syn aggregates that were identified with the monoclonal antibody 5G4 followed the same pattern as phosphorylated α-syn; *n* = 3 per group. (**C**) Overexpression of α-syn resulted in the accumulation of higher-molecular-weight α-syn species in both the soluble and insoluble fractions of midbrain homogenates (Fig. 2C). (**D**) The proteinase K (PK) resistance (5 µg/ml) of the α-syn aggregates as analysed by IHC. (**E**) Costaining of TH and α-syn (clone MJFR); *n* = 3 per group. (**F**) One month after the start of nuclear α-syn overexpression, TH was significantly reduced. WB with anti-TH antibody; *n* = 8 per group; (**G**) The levels of L-DOPA and DA in the midbrain; *n* = 8 per group; **p < 0.01; ****p < 0.0001. The error bars represent the SD.

To investigate the potential pathological changes in the AAV-α-syn-injected animals, antibodies that specifically recognize phosphorylated (clone pS129) or aggregated (clone 5G4) α-syn were used for immunohistochemical staining (IHC) analysis (Fig. 2A, B). Previous studies have shown that approximately 90% of the accumulated α-syn in the brains of PD patients is phosphorylated at serine 129. Therefore, this posttranslational modification of α-syn is considered to be a marker associated with the neuropathology of the disease [29, 30]. Similarly, the monoclonal anti-α-syn antibody 5G4 was previously shown to bind to aggregated α-syn [31]. An analysis using these antibodies showed the evident accumulation of pathological α-syn at the injection sites (Fig. 2A, B). Overexpression of α-syn^NLS^ resulted in the accumulation of the higher-molecular-weight α-syn species in both the soluble and insoluble fractions of the midbrain homogenates (Fig. 2C). Aggregates of α-syn have been reported to resist PK degradation. We found that overexpressed α-syn and α-syn^NLS^ were resistant to degradation when treated with PK for 30 min at 37 °C, but endogenous α-syn in the control group was completely degraded under these conditions (Fig. 2D). One month after injection, the mice injected with the vector that drives the expression of α-syn^NLS^ exhibited a significant decrease in the tyrosine hydroxylase (TH) levels in the midbrain (Fig. 2E, F). To determine the impact of nuclear α-syn on dopamine synthesis and storage, the levels of L-DOPA and dopamine (DA) in the midbrain were measured by HPLC. The results showed that the contents of L-DOPA and DA in α-syn group were significantly lower than those in control group, especially in nuclear α-syn group (Fig. 2G).

### Nuclear α-syn induced transcriptional dysregulation

**Fig. 3:**
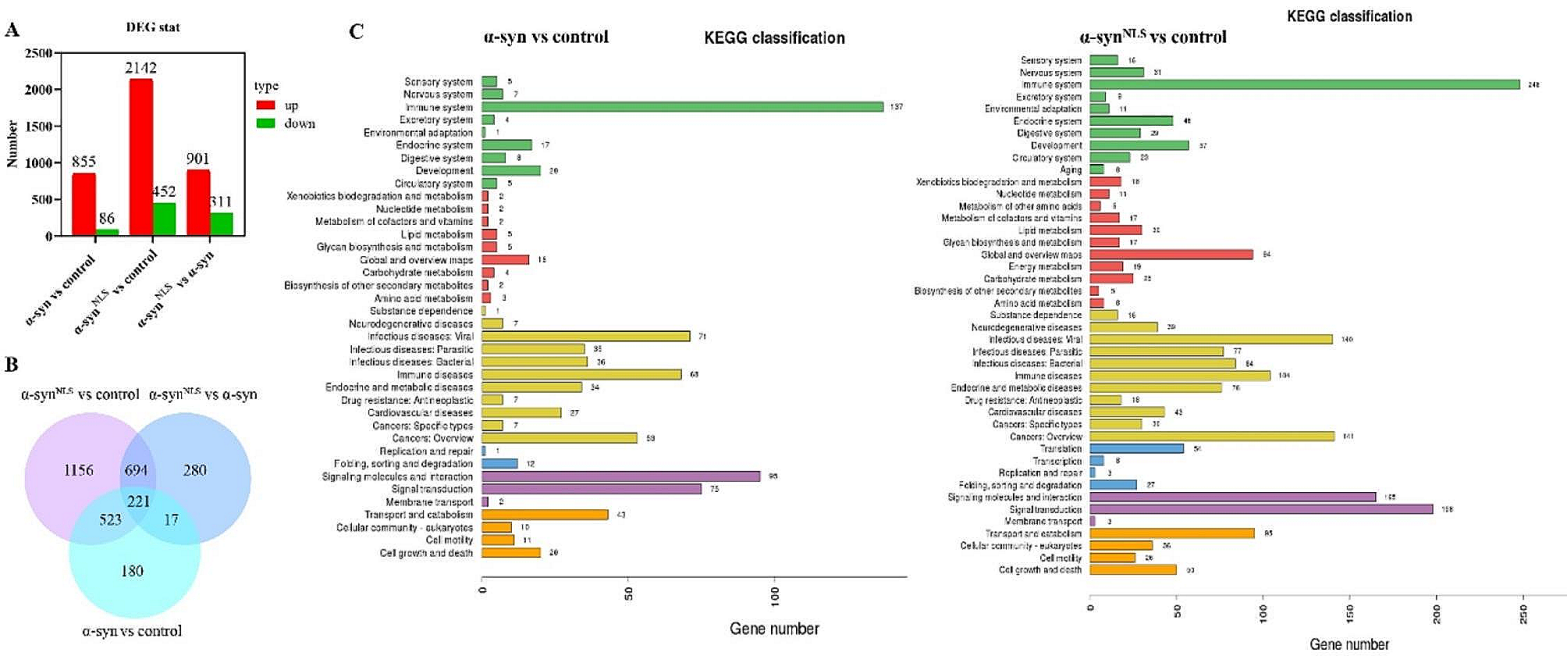
Nuclear α-syn induced strong transcriptional dysregulation (**A**) Number of DEGs in the EGFP, α-syn, and α-syn^NLS^ groups; *n* = 4 per group. (**B**) Venn diagram displaying the overlapping DEGs in the control, α-syn, and α-syn^NLS^ groups; *n* = 4 per group. (**C**) KEGG pathway analysis of the DEGs in the different groups

The impact of nuclear α-syn on the transcriptome of the midbrain was investigated using RNA-Seq. We compared the differentially expressed genes (DEGs) between the control, α-syn-overexpressing, and α-syn^NLS^-overexpressing groups (Table S1). Considering criteria of *P* ≤ 0.05 and |log_2_FC| ≥ 1 compared with the control group, 855 upregulated and 86 downregulated genes were identified in the α-syn group, and 2142 upregulated and 452 downregulated genes were identified in the α-syn^NLS^ group. A comparison between the α-syn^NLS^ and α-syn groups identified a total of 1212 DEGs, of which 901 were upregulated and 311 were downregulated in the α-syn^NLS^ group (Fig. 3A). The overlapping DEGs in the control, α-syn, and α-syn^NLS^ groups are shown in a Venn diagram (Fig. 3B). The DEGs were further investigated to determine which major pathways were affected by α-syn overexpression using Kyoto Encyclopedia of Genes and Genomes (KEGG) pathway enrichment analysis. This analysis identified the immune system pathway as one of the most predominantly affected biological processes after α-syn overexpression (Fig. 3C).

### α-syn upregulated the expression of immunity- and senescence-related genes

**Fig. 4:**
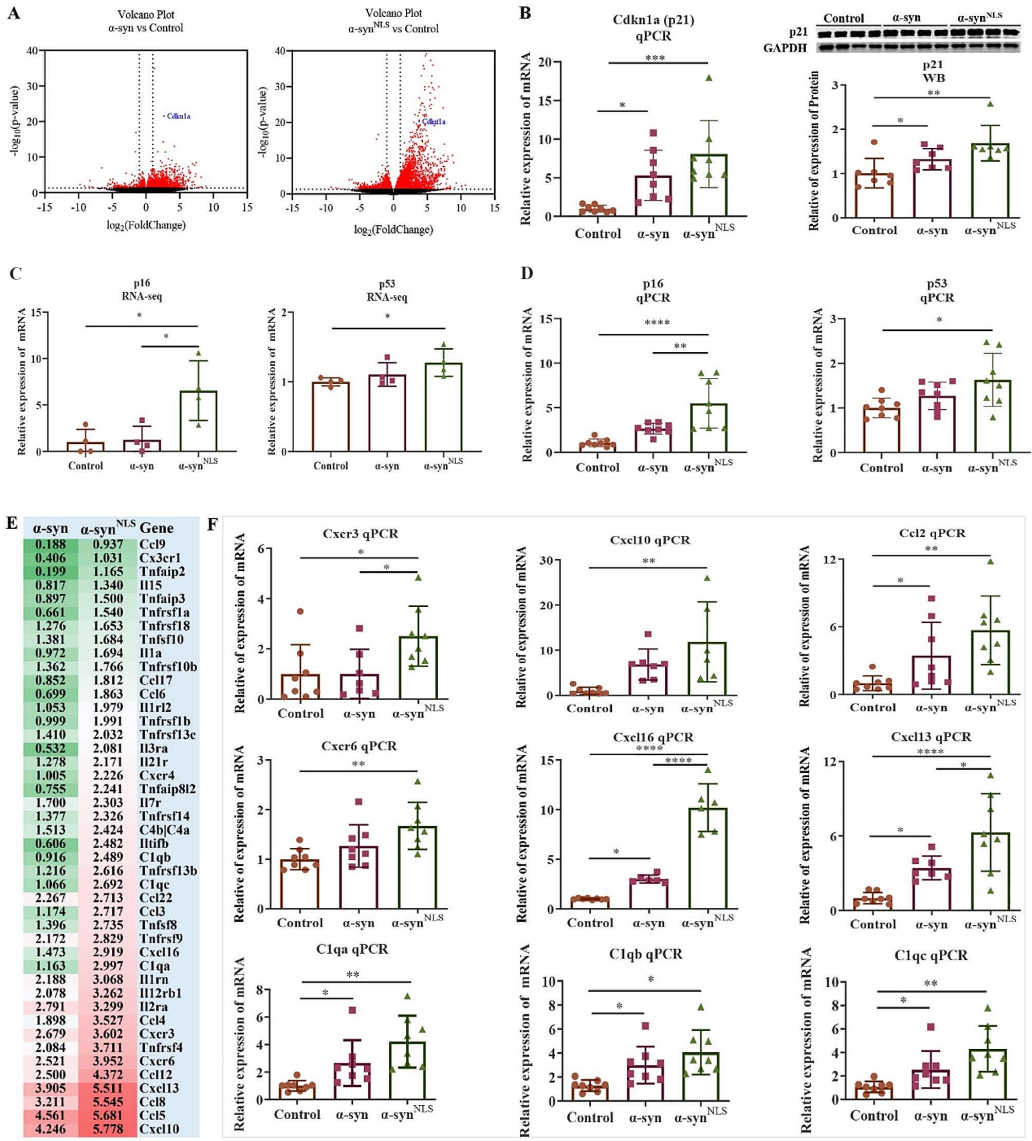
α-syn upregulated immune- and senescence-related genes (**A**) Volcano plot showing the DEGs in the α-syn group versus the α-syn^NLS^ group. Compared with the control group, α-syn overexpression, particularly in the nucleus, significantly increased the expression of *Cdkn1a* (*p21*): in the α-syn vs. control group comparison, Log_2_FC = 2.7; in the α-syn^NLS^ vs. control group comparison, Log_2_FC = 3.8; *n* = 4 per group. (**B**) Assessment of *Cdkn1a* (*p21*) expression by qPCR and WB; *n* = 8 per group. (**C**) Relative expression of *p16* and *p53* in RNA-seq data; *n* = 4 per group. (**D**) Assessment of *p16* and *p53* expression by qPCR; *n* = 8 per group. (**E**) Heatmap of the selected chemokines and inflammatory genes that were highly expressed in the α-syn and α-syn^NLS^ groups; data represent the log_2_FC values relative to the control group. (**F**) qPCR analysis confirmed a significant upregulation of *Cxcr3*, *Cxcl10*, *Cxcr6*, *Cxcl16*, *Ccl2*, *Cxcl13*, *C1qa*, *C1qb*, and *C1qc*; *n* = 6–8 per group; *p < 0.05, **p < 0.01, ***p < 0.001, ****p < 0.0001. The error bars represent the SD.

The log_2_(FC) scatter plots further confirmed that several genes were significantly upregulated in the α-syn and α-syn^NLS^ groups. This upregulation was even more robust in the α-syn^NLS^ group (Fig. 4A). The senescence marker *Cdkn1a* (p21) was significantly upregulated in the midbrain, particularly when nuclear α-syn was overexpressed [α-syn group vs. control group: log_2_(FC) = 2.7; α-syn^NLS^ group vs. control group: log_2_(FC) = 3.8]. This upregulation was confirmed by qPCR and western blotting (WB) analyses (Fig. 4B). Other senescence associated markers such as *p16* and *p53* also upregulated after nuclear α-syn overexpression in our RNA-seq data (Fig. 4C). Those results were confirmed by qPCR (Fig. 4D).

The phenomenon of ageing at the cellular level is called cellular senescence. Overexpression of α-syn, especially in the nucleus, leads to features reminiscent of cellular senescence. Senescent cells are characterized by the secretion of large amounts of inflammatory cytokines, chemokines and reactive oxygen species (ROS) [32, 33]. If senescent cells are not removed by immune cells, the persistence of the inflammatory SASP can become toxic to the surrounding cells. This mechanism is associated with a variety of age-related diseases [34]. We identified 44 chemokines and inflammatory genes that were highly expressed in the α-syn and α-syn^NLS^ groups (Fig. 4E). The upregulation of some of these genes, including *Cxcr3*, *Cxcl10*, *Cxcr6*, *Cxcl16*, *Ccl2*, *Cxcl13*, *C1qa*, *C1qb*, and *C1qc*, was confirmed using qPCR (Fig. 4F).

### α-syn induced gliosis and increased oxidative stress

**Fig. 5:**
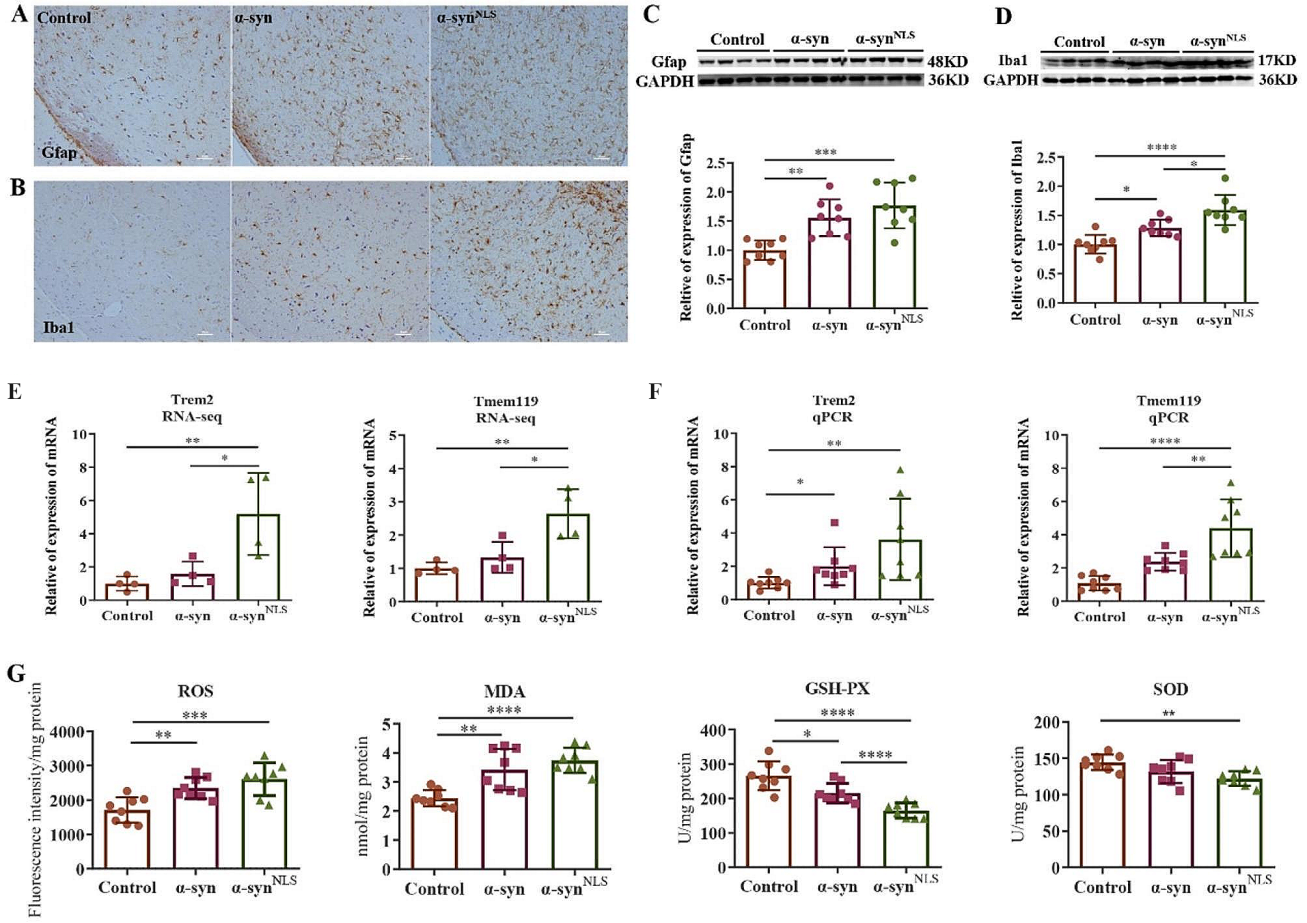
α-syn induced gliosis and increased oxidative stress in the midbrain (**A**) IHC staining of GFAP in the midbrain section. (**B**) IHC staining of Iba1 in the midbrain section. (**C**) WB with the GFAP. (**D**) WB with the Iba1. Scale bars: 50 μm. IHC: *n* = 3 per group. WB: *n* = 8 per group. (**E**) Relative expression of *Tmem119* and *Trem2* in RNA-seq data; *n* = 4 per group. (**F**) Assessment of *Tmem119* and *Trem2* expression by qPCR; *n* = 8 per group. (**G**) Levels of ROS, MDA, SOD, and GSH in the midbrain; *n* = 8 per group; *p < 0.05; **p < 0.01; ***p < 0.001; ****p < 0.0001; ns: no significant differences. The error bars represent the SD.

Neuroinflammation in the brain is mainly caused by activated glial cells (astrocytes and microglia). Increased numbers of glial cells have been reported in PD. To explore the effect of α-syn on glial cells in the brain, astrocytes and microglia were analysed using IHC staining and WB. Injection of the α-syn expression vectors into the SNc led to widespread astrocytosis and microgliosis (Fig. 5A–D). These results indicated that α-syn, especially when localized to the nucleus, obviously activated astrocytes and microglia. Two microglial markers *Tmem119* and *Trem2* also upregulated after nuclear α-syn overexpression in our RNA-seq data (Fig. 5E). Those results were confirmed by qPCR (Fig. 5F). As receptors on the surface of microglia, they are involved in processes such as inflammation, phagocytosis and aging [35–38].

Oxidative stress is an important factor in the pathogenesis of Parkinson’s disease. The relationship between α-syn accumulation and oxidative stress may form a vicious cycle and participate in disease progression. Oxidative stress occurs due to dysregulated cellular redox activity, and under these conditions, the production of ROS is greater than the ability of endogenous antioxidant enzymes and molecular chaperones to clean them. The accumulation of ROS after cellular redox imbalance causes neuronal damage [39]. Malondialdehyde (MDA) is a product of lipid peroxidation, and its level indirectly reflects the generation of free radicals during metabolism. Superoxide dismutase (SOD), through its scavenger activity, can protect cells from the toxic effects caused by free radicals. Therefore, SOD levels reflect the ability of cells to protect themselves from oxidative stress responses. Glutathione peroxidase (GSH) is a small molecule peptide composed of three amino acids that is an important antioxidant in *vivo*. GSH level is an important index to measure the antioxidant capacity of the body. Therefore, the ROS, MDA, SOD, and GSH levels in the midbrain were used to evaluate the level of oxidative stress in the different groups of mice. As shown in Fig. 5G, the overexpression of α-syn increased the levels of ROS and MDA, and decreased the levels of SOD and GSH. It indicated that α-syn accumulation in neurons may promote oxidative stress injury in the mouse brain.

### α-syn induced DNA damage, phagocytosis, and neuronal cell death

**Fig. 6:**
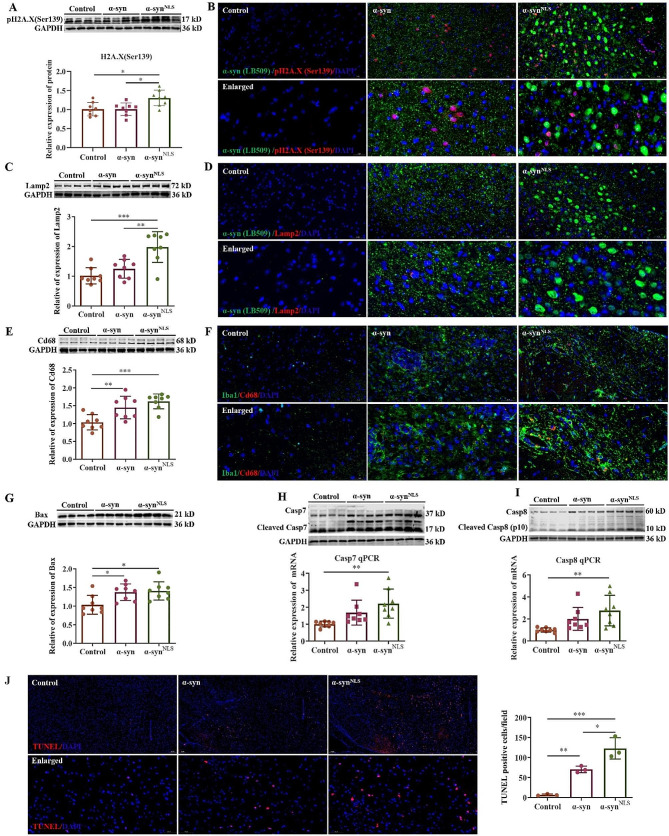
α-syn induced DNA damage, phagocytosis, and neuronal cell death (**A**) WB with an anti-phospho-histone H2A.X antibody (Ser139). (**B**) IF staining of phospho-histone H2A.X (Ser139) in midbrain sections. (**C**) WB with an anti-LAMP-2 antibody. (**D**) IF staining of LAMP-2 in the midbrain sections. (**E**) WB with an anti-CD68 antibody. (**F**) IF staining of CD68 in the midbrain sections. (**G**) WB with an anti-Bax antibody. (**H**) Assessment of Casp7 expression by WB and qPCR. (**I**) Assessment of Casp8 expression by WB and qPCR. (**J**) TUNEL staining in the midbrain. IF: *n* = 3 per group; WB: *n* = 4–8 per group; *p < 0.05; **p < 0.01; ***p < 0.001; ns: no significant differences. The error bars represent the SD.

The DNA damage marker phospho-histone H2A.X (Ser139) was used to evaluate DNA damage. As revealed by IF and WB analyses, α-syn accumulation in the midbrain was correlated with the occurrence of DNA damage (Fig. 6A, B). IF staining and WB with antibodies against the lysosome marker lysosomal-associated membrane protein-2 (Lamp-2) (Fig. 6C, D) and the microglial phagocytosis marker Cd68 (Fig. 6E, F) indicated a significant increase in the phagocytic activity in the midbrain after α-syn overexpression. These results corroborated the increase in the level of these two markers in our RNA-Seq data (Table S1). Bax (Fig. 6G), Caspase7 (Fig. 6H), and Caspase8 (Fig. 6I) levels were also significantly increased upon α-syn overexpression, which also correlated with our RNA-Seq data (Table S1). The levels of the presynaptic proteins synapsin-1 (Syn1), Syn2, and Syn3 were dramatically reduced in the α-syn and α-syn^NLS^ groups according to the RNA-Seq data (Table S1). The TUNEL staining showed that apoptotic cells increased in the midbrain after the α-syn overexpression (Fig. 6J). Together, these results indicated that α-syn accumulation, especially in the nucleus, may lead to a loss of neurons in the midbrain.

## Discussion

The distribution of α-syn in neurons is uneven, and α-syn is primarily concentrated at the synaptic nerve terminals [9]. Outeiro et al. reported that α-syn levels are nearly equal in the nucleus and cytoplasm in the embryonic stage, while α-syn is primarily located in the cytoplasm in adult brains [16]. However, the level of nuclear α-syn markedly increases under stress or in certain pathological states [21, 26]. In cultured cells, it has been reported that H_2_O_2_ induces the rapid cleavage and nuclear translocation of α-syn [11]. Oxidative stress increases the level of nuclear α-syn both in vitro and in vivo [12, 13]. Nuclear α-syn was also observed in the brains of patients with PD and dementia with Lewy body disease [13, 16] and in mouse and cellular models of these diseases [19, 21]. In the present study, we found that nucleus-targeted α-syn led to a more severe PD-like phenotype than wild-type α-syn, including a loss of dopaminergic neurons concomitant with motor impairment and the formation of α-syn inclusion bodies, as revealed by immunostaining with anti-phosphorylated α-syn (pS129) and anti-aggregated α-syn (5G4) antibodies and the result of the PK resistance test.

It has been suggested that α-syn aggregation is highly associated with the onset and progression of PD. Interestingly, high-molecular-weight α-syn species of approximately 50 kDa were observed in α-syn^NLS^-treated mice. However, the causes and precise functions of high-molecular-weight α-syn species require further clarification. Notably, nuclear α-syn seems to be involved in transcriptional regulation [16, 40–42]. Nuclear α-syn may bind histones or other nuclear factors [21, 25, 26, 43, 44] and specific DNA regions [13, 40, 45, 46]. α-Syn is prone to interacting with GC-box-like sequences and binding to DNA in a conformation-specific manner, thereby causing a conformational transition [27, 47]. These results suggest that the interaction of α-syn with nuclear proteins or DNA may alter its conformation, its function, and ultimately its transcriptomic regulation. In this study, the effect of α-syn on gene expression was evaluated using RNA-Seq. Our results showed that both α-syn and α-syn^NLS^ promoted the dysregulation of several genes, but α-syn^NLS^ had a stronger effect than α-syn. This dysregulation of gene expression primarily affected the immune response and cellular senescence pathways, and the expression of a large number of SASP-related genes was dysregulated.

We showed that α-syn induced features that are typical of cellular senescence. These included the activation of the SASP, glial activation, DNA damage, increased oxidative stress, and lysosomal dysfunction. Senescent cells express numerous inflammatory cytokines, chemokines, and ROS [48] and contribute to age-related neurodegenerative diseases, including PD [49, 50]. The symptoms of senescence observed in our model are reminiscent of those that cause the death of dopaminergic neurons in PD. The persistent presence of senescent cells in tissues causes inflammation and is harmful to surrounding cells, therefore, they contribute to aging. Signs of cellular senescence were observed after the overexpression of α-syn and especially α-syn^NLS^. Moreover, following α-syn accumulation in the nucleus, we observed high expression of the pro-senescence factor p16, p21 and p53, especially p21 was upregulated most significantly in the present study. Several studies have provided evidence of p21-dependent cell senescence in PD. Kim et al. showed that α-syn fibrils induced toxicity, which was in correlated with an increase in the levels of p21 [51]. A p21-induced senescence-like phenotype may occur in the brains of PD patients. Paul et al. found a significant increase in the p21 levels in the dopaminergic neurons of the SNc in patients with PD compared with age-matched controls [52]. *Parkin* knockout mice showed increased p21 protein levels [53]. Consistent with this finding, *Lrrk2*-mutant animals also exhibited increased p21 levels [54, 55]. These results suggest a link between p21-mediated cellular senescence and PD.

The onset and progression of PD are accompanied by increased oxidative stress and inflammatory responses [56, 57]. Oxidative stress is closely related to mitochondrial function. ROS, MDA, SOD, and GSH levels were used to evaluate the level of oxidative stress in this study. The observed increase in oxidative stress, as showed by increased ROS levels, suggested mitochondrial dysfunction. Mitochondrial dysfunction is associated with activation of neuroinflammation. Mitochondrial-derived damage-associated molecular patterns (DAMPs) are recognized by immune receptors of glial cells and aggravate neuroinflammation [58, 59]. Mitochondrial dysfunction, neuroinflammation, and oxidative stress are closely related to the pathogenesis of neurodegenerative diseases [60]. Senescent cells release numerous inflammatory cytokines, chemokines, and ROS. These factors activate glial cells that in turn release cytokines, chemokines, and ROS, which further exacerbate neuronal effects [61, 62]. Failure of immune cells to remove senescent cells creates a vicious cycle. Many chemokines have been found to be expressed by neurons. A growing body of research is elucidating the critical roles that these chemokines play in homeostasis and disease as modulators of microglial and astroglial functions [63]. Some of these chemokines and chemokine receptors, such as Cxcr3, Cxcl10, Cxcr6, Cxcl16, Ccl2, and Cxcl13, are thought to play essential proinflammatory roles [64, 65] that contribute to an inflammatory loop and subsequent neurotoxicity [63]. These genes were highly upregulated in our study. SASP determinants are usual inflammatory cytokines or immune factors that activate immune cells. In addition, the complement system plays an important role in innate immunity. The complement protein C1q is the initial responder of the classical complement pathway [66]. In the present study, the levels of three subunits of C1q (C1qa, C1qb, and C1qc) were elevated after α-syn overexpression. C1q promotes ageing-related phenotypes [67] and is involved in the development of age-dependent neurodegenerative diseases [66]. C1q is also implicated in synaptic pruning via phagocytosis [68, 69]. Our RNA-Seq analysis showed that the levels of the presynaptic proteins, Syn1, Syn2, and Syn3 (Table S1), were reduced after α-syn overexpression. We observed increased levels of the microglial phagocytosis markers Cd68 and Lamp-2. These results suggest that phagocytosis-mediated synapse loss may occur upon α-syn overexpression. In the present study, the transcriptional alterations induced by α-syn, including changes in transcription of p21 and SASP-related genes, were concomitant with gliosis, inflammation, oxidative damage, DNA damage, and lysosomal dysfunction. Phosphorylation of the histone variant H2A.X at serine 139 has been commonly used as a sensitive marker of DNA double-stranded DNA break [70, 71]. H2A.X is required for checkpoint-mediated cell cycle arrest and DNA repair following double-stranded DNA breaks [70]. In addition to its role in DNA-damage, phosphorylation of H2A.X at Ser139 is also involved in transcriptional activation [72]. It has been reported that nuclear α-syn may bind to histone proteins [25], which is likely to be involved in the regulation of transcription. The significant increase in H2A.X (Ser139) after nuclear α-syn overexpression in the present study suggests that there may be some connection between them, and it would be interesting to explore this link further in the future. Moreover, phosphorylation of H2A.X also promotes the recruitment of apoptotic proteins to DNA damage sites and thus contributes to apoptosis [73, 74]. In this study, the expression of α-syn or α-syn^NLS^ also promoted Bax, Caspase7, and Caspase8 activation, increased cellular toxicity, and increased neuronal cell death, which may eventually lead to neuronal loss and neurodegeneration.

The current studies on the toxicity associated with nuclear α-syn are contradictory. Most report increased neurotoxicity [13, 15, 21], while others report decreased neurotoxicity [26, 75]. These discrepancies suggest that α-syn may play different roles in the nucleus in response to different stress conditions or specific molecular factors [26]. In this study, AAV2/9 vectors were used to express α-syn, resulting in its relatively high expression (approximately 1.5 × 10^13^ vector genomes/ml). The toxicity of α-syn is known to be dose-dependent [76]. This high dose may explain the discrepancies between our results and some studies showing that nuclear α-syn may exert a protective effect.

**Fig. 7:**
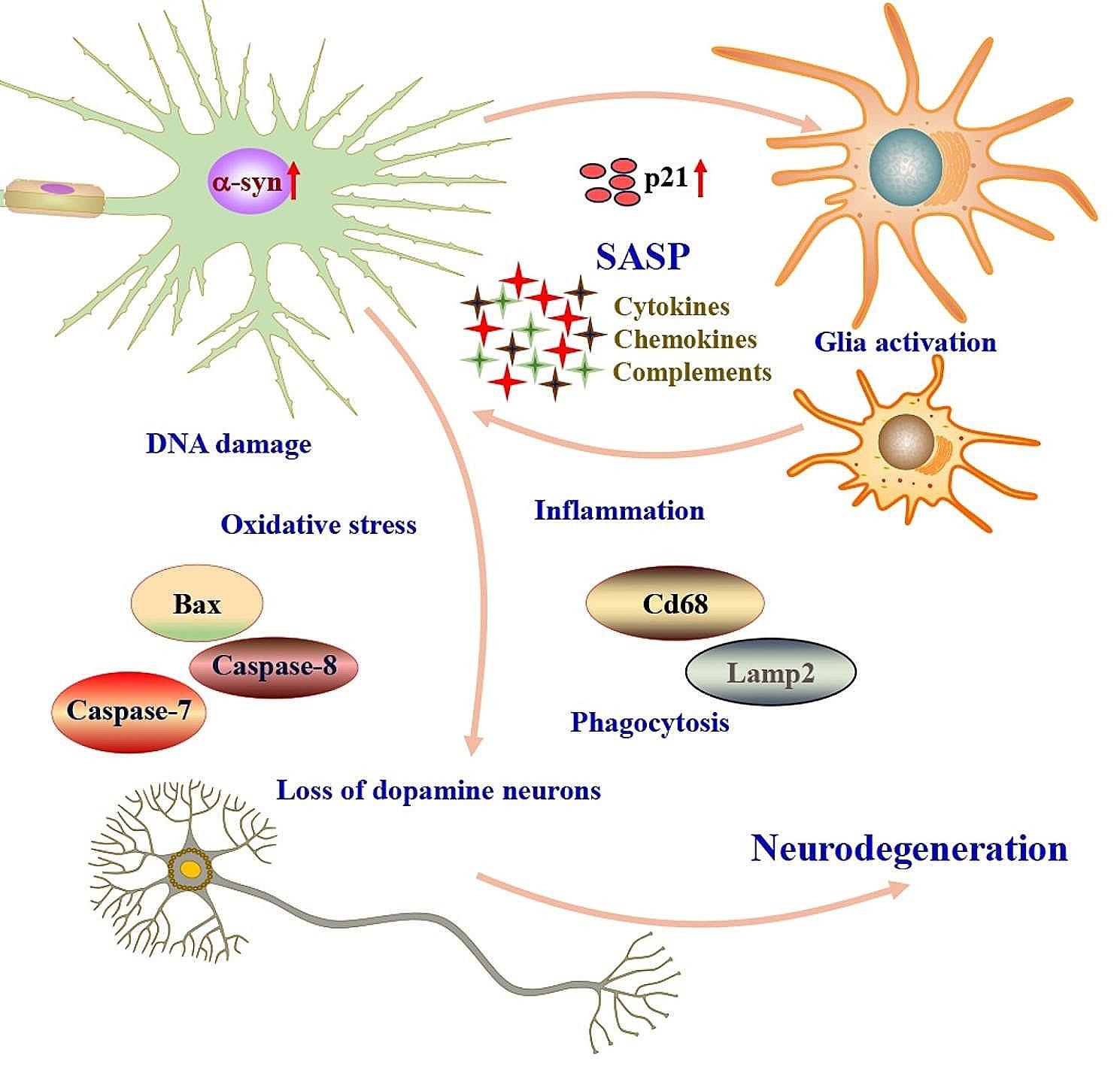
Diagram summarizing the nuclear α-syn-mediated neurodegeneration Nuclear α-syn induced cell senescence accompanied by high expression of p21 and SASP-related factors. Senescent cells activate glial cells, and glial cells may, in turn, further promote cell senescence. This process leads to synapse loss and neuronal cell death by promoting inflammation, oxidative stress, and phagocytosis, thus contributing to PD pathogenesis. These schematic illustrations were drawn using ScienceSlides (http://www.scienceslides.com)

In summary, we found that nuclear α-syn is a critical mediator of PD. Our results indicated that the increased expression of α-syn in the nucleus may exacerbate the progression of PD. Increased nuclear α-syn induced cell senescence accompanied by the high expression of p21 and SASP-related factors increases glial cell activation, and glial cells may in turn further promote cell senescence. This process leads to synapse loss and neuronal cell death by promoting inflammation, oxidative stress, and phagocytosis, thus contributing to PD pathogenesis (Fig. 7). This study demonstrated an important role of nuclear α-syn in mediating PD and provides a new perspective on the development of PD. Further studies are required to shed light on the role of nuclear α-syn in the pathophysiology of PD.

## Materials and methods

### Animals

C57BL/6J mice at 10–11 weeks of age (18–25 g; Vital River Animal Technology Co. Ltd., Beijing, China) were housed in groups of two animals per cage in a room with a 12/12-h light/dark cycle and constant temperature (23 °C ± 2 °C), and the mice were given *ad libitum* access to food and water. All of the mice were allowed to acclimate to the housing conditions for two weeks prior to the beginning of the experiments. This study was conducted in accordance with the recommendations of the Yunnan Province Experimental Animal Management Association. The protocol was approved by the Experimental Animal Ethic Committee of the Institute of Medical Biology of the Chinese Academy of Medical Sciences.

### Stereotaxic surgery

C57BL/6 male mice were stereotaxically injected with AAV2/9 vectors carrying different coding sequences. The viral vector AAV2/9-α-syn, which drives the expression of the human *SNCA* gene, or AAV2/9-α-syn^NLS^, which drives the expression of the human α-syn gene with an NLS, was stereotaxically injected into the SNc of the mice. The mice in the control group were injected with AAV2/9 expressing enhanced green fluorescent protein (EGFP). Prior to surgery, the mice were anaesthetized with 50 mg/kg pentobarbital sodium. During surgery, AAV-hSyn-α-syn (α-syn group), AAV-hSyn-α-syn^NLS^ (α-syn^NLS^ group), or AAV-hSyn-EGFP (control group) was injected into the SNc in volumes of 2 µl per side. The coordinates were as follows: anterior–posterior, − 3.1 mm; medial–lateral, ± 1.3 mm; and dorsal–ventral, − 4.5 mm. Behavioural tests were performed one month after injection. Recombinant AAV2/9 that carried α-syn, α-syn^NLS^, or EGFP was purchased from Taitool Bioscience (Shanghai Taitool Bioscience Co. Ltd., China). The final titre of each rAAV was approximately 1.5 × 10^13^ vector genomes/ml.

### Rotarod test and pole descent test

The rotarod test was conducted using an accelerating rotarod (Shanghai, YLS-4 C) to estimate the balance and motor coordination of the mice. Briefly, the mice were placed on 3-cm diameter rods, and the time it took each animal to maintain its balance was measured. The animals received two days of training prior to beginning the experiments. The speed of the rotarod accelerated from 0 to 40 rpm over 2 min and was maintained at 40 rpm for 3 min. The latency to falling off the rod was recorded. Trials were performed in triplicate. The mean latency to falling off the rod was used for the analysis.

A 50-cm long and 1-cm diameter pole was wrapped in a non-adhesive shelf liner to facilitate grip and placed in a housing cage. The animals received two days of training in descending from the top of the pole to the housing cage. On the test day, the animals were placed head down on the top of the pole, and the time required to reach the bottom of the housing cage was recorded. Timing began when the researcher released the animal and ended when one hind limb reached the cage. Trials were performed in triplicate.

### RNA-seq

After the behavioural tests, total RNA was extracted from the midbrain, and a cDNA library was prepared according to the standard instructions of Illumina (TruSeq Stranded RNA LT Guide). An Agilent 2100 bioanalyzer was used to evaluate the concentration and size distribution of the cDNA library prior to sequencing with the Illumina HiSeq 2500 system. High-throughput sequencing was performed strictly following the manufacturer’s instructions (Illumina HiSeq 2500 User Guide). The raw reads were filtered by Seqtk prior to genome mapping with Tophat (version: 2.0.9). The gene fragments were counted using HTSeq followed by the trimmed mean of the M values (TMM) normalization. Differentially expressed genes (DEGs) were defined as genes with |log_2_(fold change [FC])| ≥ 1 and *P* ≤ 0.05 using DESeq2 software.

### Immunohistochemistry and immunofluorescence

The mice were deeply anaesthetized using a pentobarbital injection (50 mg/kg) and perfused with 0.1 M PBS followed by 4% paraformaldehyde. The brains were fixed in 4% paraformaldehyde overnight and then embedded in paraffin. The paraffin-embedded tissues were sectioned at 4 μm using a microtome (Leica, RM2235, Germany). After antigen retrieval using sodium citrate buffer in a microwave oven and a 15-min incubation with 3% H_2_O_2_ to block endogenous peroxidase activity, the sections on slides were incubated with appropriate antibodies overnight at 4 °C for IHC staining. The antibodies used in this study are listed in Table 1. After washing three times with PBS, the sections were incubated with biotin-conjugated secondary antibodies (SP Rabbit & Mouse HRP Kit, CW2069) for 1 h at room temperature and visualized using 3,3-diaminobenzidine (DAB). To assess proteinase K (PK)-resistant α-syn aggregates, the sections were pretreated with PK (5 µg/ml) at 37 °C for 30 min. Then, the sections were analysed using IHC staining with a monoclonal antibody (BD, 610,787) against both mouse and human α-syn.

**Table 1:** Antibodies used in this study

Primary antibodies	Type	Source	WB	IF/IHC
Anti-hα-syn (MJFR1)	Rabbit mono	Abcam (ab138501)	1/1000	1/200
Anti-hα-syn (LB509)	Mouse mono	Abcam (ab27766)	1/1000	1/200
Anti-α-syn (BD)	Mouse poly	BD (610,787)	—	1/500
Anti-α-syn (pS129)	Mouse mono	Wako (015-25191)	—	1/200
Anti-α-syn (5G4)	Mouse mono	Merck (MABN389)	—	1/500
Anti-TH	Rabbit poly	ABclonal (A12756)	1/1000	1/1000
Anti-p21	Rabbit poly	CST (2947)	1/1000	—
Anti-Gfap	Mouse mono	Sigma (G3893)	—	1/1000
Anti-Gfap	Rabbit poly	Abcam (ab7260)	1/1000	—
Anti-Iba1	Rabbit poly	Wako (016-20001)	1/1000	—
Anti-Iba1	Mouse mono	Merck (MABN92)	—	1/200
Anti-H2A.X (Ser139)	Rabbit poly	CST (9718)	1/1000	1/500
Anti-Cd68	Rabbit poly	CST (19,589)	1/1000	1/200
Anti-Lamp2	Rabbit poly	ABclonal (A14017)	1/1000	1/200
Anti-Bax	Rabbit poly	Proteintech (50599-2-Ig)	1/1000	—
Anti-GAPDH	Rabbit poly	Proteintech (10494-1-AP)	1/5000	—

For IF staining, endogenous peroxidase activity blocking step was omitted. After incubation with primary antibodies at 4 °C overnight, the slides were washed three times with PBS and incubated with Alexa Fluor-conjugated secondary antibodies for 1 h at room temperature. Next, the sections were washed three times with PBS and mounted with a coverslip in a Fluoroshield mounting medium containing 4′,6-diamidino-2-phenylindole (DAPI; Abcam, ab104139). Images were captured using a panoramic MIDI digital scanner (3DHISTECH, Hungary). The antibodies used in this study are listed in Table 1. Four types of anti-α-syn antibodies were used for IF/IHC: rabbit anti-human α-syn (clone MJFR1) and mouse anti-human α-syn (clone LB509) were used specifically to detect human α-syn; mouse anti-α-syn (BD) was used to detect both mouse and human α-syn; anti-α-syn (clone pS129) was specific for the phosphorylated forms of α-syn; and anti-α-syn (5G4) was specific for the aggregate forms of α-syn. The secondary antibodies were Alexa Fluor 594-conjugated goat anti-rabbit (Invitrogen, A-11,012, 1:500), Alexa Fluor 488-conjugated goat anti-mouse (Invitrogen, A-11,001, 1:500), Alexa Fluor 488-conjugated goat anti-rabbit (Invitrogen, A-11,034, 1:500), and Alexa Fluor 594-conjugated goat anti-mouse (Invitrogen, A-11,005, 1:500). Images were captured using a panoramic MIDI digital scanner (3DHISTECH, Hungary). At least three brain sections from each group were analysed.

### Western blotting

The samples were homogenized in radioimmunoprecipitation assay (RIPA) lysis buffer (CW2333, CWBIO, China) supplemented with 2 mM phenylmethylsulfonyl fluoride (PMSF) and a protease inhibitor cocktail (539,131, Millipore, USA) until there were no more visible pieces of tissue. The homogenates were transferred to new tubes and centrifuged at 20,000 × *g* for 15 min at 4 °C. The protein concentrations of the samples were measured using the bicinchoninic acid (BCA) method (BCA Protein Assay Kit, CW0014S, CWBIO). The protein samples were separated on Criterion TGX Stain-Free gels (Bio-Rad) for 120 min at 85 V. The proteins were then transferred to nitrocellulose membranes (66,485, Biolab, USA) for 5 min using the Trans-Blot Turbo Transfer System (Bio-Rad). After transfer, the membranes were blocked with a 5% nonfat milk buffer for 1 h at room temperature under gentle agitation. The membranes were then incubated with primary antibodies overnight at 4 °C. The antibodies used in this study are listed in Table 1. After incubation with primary antibodies, the membrane was washed three times with Tris-buffered saline containing 0.1% Tween 20 (TBST) for 15 min. The membranes were then incubated with IRDye 800CW-conjugated goat anti-rabbit or anti-mouse secondary antibodies (1:10,000, 926-32211 or 926-32210, LI-COR, USA) or IRDye 680RD-conjugated goat anti-rabbit or anti-mouse secondary antibodies (1:10,000, 926-68071 or 926-68070, LI-COR, USA) for 1 h at room temperature. Next, the membranes were washed three times with TBST for 15 min, and images were acquired using an Odyssey Imager (LI-COR, USA). The signal intensities were quantified using Odyssey software (version 3.0, LI-COR, USA).

Soluble and insoluble α-syn was isolated as previously described [77]. Briefly, each midbrain sample was homogenized in 500 µL of lysis buffer (50 mM Tris, pH 7.4, 150 mM NaCl, and 5 mM EDTA) containing a protease inhibitor cocktail (Millipore, America). Triton X-100 was added to the homogenates at a final concentration of 1%. After 30 min of incubation on ice, the homogenates from each sample were centrifuged (20,000 × *g*) at 4 °C for 1 h. The supernatants from each sample were transferred to a fresh tube and designated as “soluble α-syn.” The pellets were solubilized in a lysis buffer containing 2% SDS, and following incubation on ice for 30 min; these samples were designated “insoluble α-syn”. The soluble and insoluble α-syn was subsequently immunoblotted as described above.

### Neurochemical analysis

Mouse brain tissue samples for this study were analyzed for L-DOPA and dopamine (DA) using the Shimadzu LC-20 A Prominence UPLC System (Shimadzu, Japan). All separations were performed out using a Diamonds C18 (150 mm × 4.6 mm × 5 μm) at a column temperature of 35 °C and a flow rate of 1 mL/min. The mobile phase consisted of sodium citrate buffer (pH 3.8) and methanol (87:13, v/v). The sodium citrate buffer consisted of 10 mM citric acid, 25 mM NaH_2_HPO_4_, 25 mM EDTA, and 2 mM of 1-heptane sulfonic acid. Brain samples were homogenized in 0.2 M perchloric acid and centrifuged at 12,000 g for 5 min. The supernatant was filtered through 0.22-mm nylon filters before injection in the UPLC. The chromatographic process used equal concentrations and elutions and was maintained at a temperature of 10 °C during the entire analytical process, with a 10-µL injection volume.

### Quantitative real-time PCR

Total RNA was extracted from mouse brain using TRI Reagent (T9424, Sigma-Aldrich, USA) according to the manufacturer’s instructions. The concentration and quality of the RNA were determined by measuring the absorbances at 260 and 280 nm using a spectrophotometer. Values between 1.8 and 2.0 were indicative of high-quality RNA. Next, 1 µg of the total RNA from each sample was reverse transcribed to cDNA using the Eastep RT Master Mix (5×) Kit (LS2054, Promega, USA). Quantitative real-time PCR (qPCR) was performed using the SYBR Green method with Eastep qPCR Master Mix (2×) (LS2068, Promega, USA) in a CFX96TM Real-Time PCR Detection system (Bio-Rad, USA) according to the manufacturer’s instructions. The expression of each target gene was analysed in triplicate. The mRNA levels of *Cdkn1a (p21), Cdkn2a (p16)*, *Trp53 (p53)*, *Cxcr3*, *Cxcl10*, *Cxcr6*, *Cxcl16*, *Ccl2*, *Cxcl13*, *C1qa*, *C1qb*, *C1qc, Tmem119, Trem2, Caspase7 and Caspase8* were quantified using the comparative Ct (2^−ΔΔCt^) method, with *Gapdh* mRNA as the internal control. Primer sequences were obtained from PrimerBank. The results are presented as FC values. The primers are listed in Table 2.

**Table 2:** Primers used in this study

Gene	Primer F	Primer R
*Cdkn1a*	CCACAGCGATATCCAGACATTC	GAAGTCAAAGTTCCACCGTTCTC
*Cdkn2a*	GCTCAACTACGGTGCAGATTC	GCACGATGTCTTGATGTCCC
*Trp53*	CCCCTGTCATCTTTTGTCCCT	AGCTGGCAGAATAGCTTATTGAG
*Cxcr3*	TCTCCCTACGATTATGGGGAAAA	GGTTCTGTCAAAGTTCAGGCT
*Cxcl10*	CCAAGTGCTGCCGTCATTTTC	TCCCTATGGCCCTCATTCTCA
*Ccl2*	TAAAAACCTGGATCGGAACCAAA	GCATTAGCTTCAGATTTACGGGT
*Cxcr6*	GAGTCAGCTCTGTACGATGGG	TCCTTGAACTTTAGGAAGCGTTT
*Cxcl16*	CCTTGTCTCTTGCGTTCTTCC	TCCAAAGTACCCTGCGGTATC
*Cxcl13*	GGCCACGGTATTCTGGAAGC	ACCGACAACAGTTGAAATCACTC
*C1qa*	CCAGGAGAGTCCATACCAGAA	GTCCCACTTGGAGATCACTTG
*C1qb*	GGCAACCTGTGTGTGAATCTC	CTCTAGCTTCAAGACTACCCCA
*C1qc*	AGAAGCACCAGTCGGTATTCA	TGCGATGTGTAGTAGACGAAGTA
*Tmem119*	TCTTCCGGCAGTACGTGATG	CGGCGCAGACTATGAACATGA
*Trem2*	CTGGAACCGTCACCATCACTC	CGAAACTCGATGACTCCTCGG
*Caspase7*	AAGACGGAGTTGACGCCAAG	CCGCAGAGGCATTTCTCTTC
*Caspase8*	CAACTTCCTAGACTGCAACCG	TCCAACTCGCTCACTTCTTCT
*Gapdh*	TGTGTCCGTCGTGGATCTGA	TTGCTGTTGAAGTCGCAGGAG

### Quantification of reactive oxygen species, superoxide dismutase, malondialdehyde, and glutathione peroxidase levels

Since oxidative stress is believed to be an important contributor to the neurodegenerative process occurring that occurs in PD [78], the levels of ROS, SOD, MDA, and GSH-PX were measured using kits from the Nanjing Jiancheng Bioengineering Institute (Nanjing, China). ROS levels were quantified by measuring the fluorescence of 2,7-dichlorofluorescin diacetate (DCFH-DA; E004-1-1, Njjcbio, China). Tissues were homogenized in phosphate-buffered saline (PBS) and incubated with 100 µM DCFH-DA for 1 h at 37 °C in the dark. The fluorescence intensities were quantified using a Multimode Microplate Reader (Thermo Scientific Varioskan LUX, USA) at an excitation wavelength of 485 nm and an emission wavelength of 545 nm. Mouse midbrain tissues were homogenized on ice and processed using a total SOD activity detection kit (WST-8 method; A001-3-2, Njjcbio, China), an MDA assay kit (TBA method; A003-1-2, Njjcbio, China), and a GSH-PX assay kit (colorimetric method; A005-1-2, Njjcbio, China) according to the manufacturer’s instructions.

### Tunel staining

Apoptosis in the midbrain was determined using the terminal deoxynucleotidyl transferase-mediated YF594-dUTP nick-end labeling (TUNEL) method following the manufacturer’s protocol (YF594 TUNEL Assay Apoptosis Detection Kit; US Everbright Inc, China, T6014). The nuclei were stained with DAPI.

### Statistical analysis

GraphPad Prism software (GraphPad Prism 8) was used for the statistical analyses, and the images were processed using Adobe Photoshop CS6. One-way analysis of variance (ANOVA) was performed for multiple-group comparisons. All the data are shown as the mean ± standard deviation (SD).

### Electronic supplementary material

Below is the link to the electronic supplementary material.

Supplementary Material 1

## Data Availability

Raw RNA-seq data files are available at National Genomics Data Center (NGDC), China National Center for Bioinformation under the accession number CRA010130 (Shared URL: https://ngdc.cncb.ac.cn/gsa/s/2rK62BRd).

## Abbreviations

PD: Parkinson’s disease
α-syn: alpha-synuclein
AAV: adeno-associated virus
NLS: nuclear localization sequence
SASP: senescence-associated secretory phenotype
IF: immunofluorescence staining
SNc: substantia nigra compacta
IHC: immunohistochemical staining
WB: western blotting
TH: tyrosine hydroxylase
ROS: reactive oxygen species
SOD: superoxide dismutase
MDA: malondialdehyde
GSH: glutathione peroxidase
LAMP-2: lysosomal-associated membrane protein-2
DAMPs: Mitochondrial-derived damage-associated molecular patterns
KEGG: Kyoto Encyclopedia of Genes and Genomes
EGFP: enhanced green fluorescent protein
